# CRISPR-mediated phage resistance and the ghost of coevolution past

**DOI:** 10.1098/rspb.2010.0055

**Published:** 2010-03-17

**Authors:** Pedro F. Vale, Tom J. Little

**Affiliations:** Institute of Evolutionary Biology, University of Edinburgh, The Kings Buildings, West Mains Road, Edinburgh EH9 3JT, UK

**Keywords:** bacteria, phage, coevolution, evolution, epidemiology, resistance

## Abstract

The past is never dead. It's not even past

William Faulkner (1951)

Bacteria can acquire heritable immunity to viral (phage) enemies by incorporating phage DNA into their own genome. This mechanism of anti-viral defence, known by the acronym CRISPR, simultaneously stores detailed information about current and past enemies and the evolved resistance to them. As a high-resolution genetic marker that is intimately tied with the host–pathogen interaction, the CRISPR system offers a unique, and relatively untapped, opportunity to study epidemiological and coevolutionary dynamics in microbial communities that were previously neglected because they could not be cultured in the laboratory. We briefly review the molecular mechanisms of CRISPR-mediated host–pathogen resistance, before assessing their potential importance for coevolution in nature, and their utility as a means of studying coevolutionary dynamics through metagenomics and laboratory experimentation.

## 1. Introduction

Evolution is explicitly about change over generations, and that can make it difficult to study when generation times are long, or when organisms are difficult to routinely assay. It is therefore convenient when evolution leaves behind traces of its activity. Fossils record the past, but offer a poor record of evolution at the molecular level. Fortunately, evidence of the past is often imprinted upon DNA sequences. For example, sequence comparisons can be used to estimate the proportion of differences between species that are driven by natural selection (as opposed to random walks of neutral variation), and even to estimate the speed at which adaptation has proceeded (Nielsen 2005). Such analyses have indicated that, perhaps more than any other class of genes, those of immune systems tend to evolve adaptively and rapidly (Bustamante *et al*. 2005). This suggests that host–parasite coevolution plays a central role in the maintenance of biological diversity (Buckling & Rainey 2002*a*; Laine & Tellier 2008). Still, most studies focus on the past evolutionary record of host defence genes, or, separately, of parasite infectivity genes, and thus address coevolution indirectly. Recently, a remarkable mechanism has been described that may provide, in the DNA sequence of a single organism, a detailed molecular record of coevolution. Clustered regularly interspaced short palindromic repeats (CRISPRs) are arrays of prokaryotic DNA sequences that mediate a form of acquired immunity to specific viral pathogens (Sorek *et al*. 2008; van der Oost *et al*. 2009)—something usually associated with vertebrate defence systems—and in the process provide a record of coevolution past. The study of this defence mechanism has already revealed some fascinating molecular biology, but relatively untapped is the potential to explore the ongoing warfare between hosts and their phage pathogens in natural environments.

## 2. Acquired, specific and heritable bacterial resistance to viral pathogens

**Figure 1.**
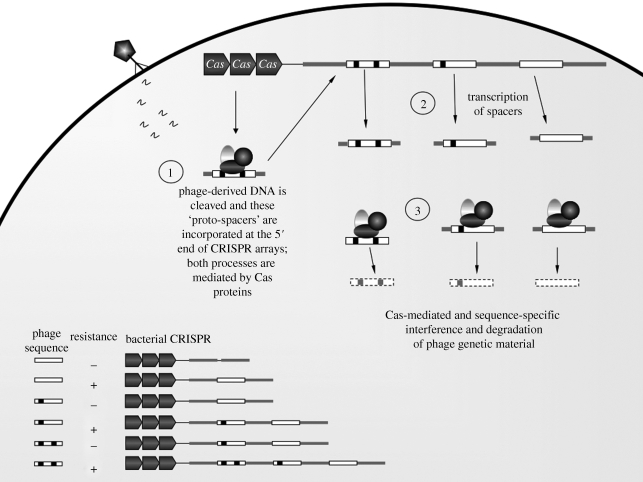
A schematic representation of CRISPR-mediated phage resistance. CRISPR-associated (Cas) proteins play an important role in the initial recognition of phage genetic material and incorporating these proto-spacers in the CRISPR array (1). Once incorporated, these spacers are then transcribed (2) and used as templates to target homologous phage sequences within the bacterial cell, again mediated by Cas protein complexes (3). The bottom left of the figure illustrates the specificity of CRISPR-mediated resistance. While resistance to a specific phage genotype can be acquired by incorporating a spacer derived from that genotype, point mutations in the phage (represented by the black squares) are sufficient to evade resistance. Hosts are only able to resist this mutant by incorporating a phage-derived spacer containing the new mutation. This could lead to an ongoing ‘arms race’, with hosts incorporating more spacers in response to increasing phage mutation accumulation. The order in which these spacers are incorporated also provides a sequential record of past phage infections.

Viruses that infect bacteria (bacteriophages) are ubiquitous in natural bacterial communities (Abedon 2008), and in some environments outnumber their bacterial hosts nearly tenfold (Suttle 2005). Bacteria have evolved a number of phage-resistance mechanisms, such as adsorption blocking, restricting the injection of phage genetic material and some abortive infection mechanisms (Sturino & Klaenhammer 2006). An additional mechanism has recently been added to this list: CRISPRs are present in bacterial and archaeal genomes, and are made up of arrays of highly conserved 24- to 47-bp repeats, separated by variable, often unique spacer sequences, derived from foreign replicons such as phage or plasmids (figure 1; Sorek *et al*. 2008). In a series of elegant experiments (Barrangou *et al*. 2007; Brouns *et al*. 2008; Deveau *et al*. 2008), it was recently shown that: (i) bacterial cells incorporate phage genetic material into CRISPRs as spacers; (ii) acquiring these spacers renders bacterial hosts resistant towards phage that carry the incorporated sequence; (iii) removal of these spacers results in the loss of this resistance; and (iv) single mutations at specific short motifs in the phage genome are sufficient to evolve counter-resistance (figure 1). This potential for reciprocal adaptation to host resistance and pathogen infectivity marks CRISPRs as a potential molecular mechanism for coevolution.

An integral part of this anti-phage defence mechanism is the action of CRISPR-associated (Cas) proteins. Cas proteins are usually found adjacent and upstream of CRISPR arrays, and show high sequence and structural similarity to proteins with endonuclease, and DNA- and RNA-binding functions (Jansen *et al*. 2002; Wiedenheft *et al*. 2009). Because of this feature, they have been thought to function similarly to RNA-interference (RNAi) genes (reviewed in Marraffini & Sontheimer 2010*a*), which themselves mediate a rapidly evolving antiviral defence in eukaryotes (Ding *et al*. 2004; Obbard *et al*. 2009). However, despite some similarities, their protein machineries are distinct (Makarova *et al*. 2006) and, unlike RNAi, CRISPR–Cas complexes appear to bind almost exclusively to DNA, suggesting that RNAi and Cas proteins share little phylogenetic relation (Mojica *et al*. 2009).

The molecular details are still being unravelled, but the anti-viral action of the CRISPR–Cas complex appears to proceed in three distinct stages (figure 1; reviewed in Marraffini & Sontheimer 2010*a*). In the initial stage of viral invasion, complexes of Cas proteins target and cleave short recognition motifs in the phage genome (Deveau *et al*. 2008; Mojica *et al*. 2009), and incorporate these ‘proto-spacers’ into the host genome at the 5′ end of the CRISPR locus (Barrangou *et al*. 2007). These short (23–30 bp) incorporated ‘spacers’ are then transcribed (stage 2) as CRISPR RNAs (CrRNAs) that contain the spacer sequence flanked on either side by partial repeat sequences (Brouns *et al*. 2008), resulting in large-scale amplification of that specific sequence. It is still unclear whether all spacers (e.g. Lillestol *et al*. 2009) or only the most recently incorporated (e.g. Brouns *et al*. 2008) are transcribed during this step. The final stage involves interference with phage-derived sequences, again mediated by Cas-protein complexes, whereby CrRNAs serve as templates to target conserved viral motifs in subsequent infections (Brouns *et al*. 2008). How do CRISPRs avoid targeting and degrading their own phage-derived CRISPR spacer? Viral targets are identified by direct Watson–Crick pairing, and viral degradation seems to be triggered by mismatches at specific positions between the viral sequence and the repeat sequences flanking the phage-derived spacer (Marraffini & Sontheimer 2010*b*). Therefore, CRISPRs are truly a microbial immune system (Horvath & Barrangou 2010), allowing strain-specific resistance to be acquired, a memory of past infections that permits resistance against future encounters, while assuring host integrity through self/non-self discrimination.

How common is CRISPR-mediated defence in nature? Recently, identifying and characterizing the diversity of CRISPR–Cas complexes in the wild has been made possible by high-throughput next-generation sequencing technologies (Mardis 2008). Using a metagenomic approach (Hugenholtz & Tyson 2008), environmental samples can be entirely sequenced and screened with recently developed CRISPR- and Cas-finding algorithms (Haft *et al*. 2005; Bland *et al*. 2007; Edgar 2007; Grissa *et al*. 2007, 2008*a*), which are now freely available through web-based resources (Grissa *et al*. 2008*b*; also see CRISPR databases at http://crispr.u-psud.fr and http://crispi.genouest.org). These ever-growing interactive databases find CRISPR–Cas complexes in roughly 40 per cent of bacterial and nearly 90 per cent of archaeal genomes tested so far. Up to 45 Cas protein families have been described based on amino acid sequences (Haft *et al*. 2005). Two of these proteins, Cas 1 and Cas 2, seem to be present among all CRISPR systems described to date, and so offer potential universal markers of CRISPR-mediated defence systems in microbes (Makarova *et al*. 2006; Sorek *et al*. 2008). The crystal structure of Cas 1 indicates that it has a metal-dependent DNase activity, so it is thought to be involved in the initial recognition and acquisition of viral motifs (Wiedenheft *et al*. 2009), whereas Cas 2 has been shown to cleave single-stranded RNA within U-rich regions.

Beyond mechanisms, CRISPRs offer a unique window into the history of bacteria–phage warfare, as exposure to a specific combination of phage strains leaves behind a unique set of spacers in the host bacterial genome that simultaneously represents both infective phage strains that have been prevalent in the environment and the specific resistance bacteria have acquired. Because phage-derived spacers are incorporated at the CRISPR 5′ leader sequence (Barrangou *et al*. 2007), the ordered sequence of spacers essentially gives a temporal record of the infection history in that bacterial population. Hence, CRISPRs offer a high-resolution method for molecular typing of bacterial hosts and their pathogens based on the unique CRISPR signature. Such record-keeping of both host and pathogen genetic variation over time is unparalleled in any other host–pathogen system. Therefore, CRISPRs might be especially informative in determining the history of phage host ranges, which would be impossible to determine from other resistance mechanisms. Below we highlight how CRISPRs offer a valuable tool for studies of coevolution, both in the laboratory and, importantly, in the wild.

## 3. Patterns of adaptation in space and time

Bacterial CRISPRs offer a unique opportunity to address questions of coevolutionary dynamics in natural populations, because only bacteria, which contain a record of both their evolution and phage evolution in their CRISPR signatures, need to be sampled. Crucially, metagenomic analyses can be carried out without the need to culture bacteria in the laboratory, which means that CRISPR-mediated coevolution can be studied in non-cultivable bacteria. This approach potentially permits coevolutionary dynamics to be studied in detail, either by geographic sampling to identify patterns of local adaptation, or by temporal sampling where hosts and phage are directly and simultaneously tracked over time.

One of the tenets of host–parasite coevolution is that antagonistic selection proceeds through local adaptation of pathogens to common host genotypes, followed by counter-adaptation of hosts (Hamilton 1993; Woolhouse *et al*. 2002). Accordingly, the most common method for inferring coevolution in wild populations is to sample over geographic space and test whether hosts and parasites are locally adapted (Greischar & Koskella 2007). Recently, this approach has been extended to analyses of CRISPR sequences from environmental samples. For example, Kunin *et al*. (2008) examined samples from two geographically distant (USA and Australia) sludge bioreactors. Focusing on the dominant, and as yet uncultured, microbial species (*Candidatus* Accumulibacter phosphatis or CAP), analysis of CRISPR arrays gave strikingly different results from a thorough analysis of the rest of the bacterial genome. Specifically, analysis of CRISPR arrays revealed that there were no common spacer sequences between the two populations, while there was very little divergence at 48 other loci. To reconcile these differences, the authors hypothesized that CAP strains disperse widely to become genetically homogeneous (hence to very low divergence found in most of the genome), but differences in exposure to local phage populations lead to rapid divergence of CRISPR loci. By sequencing the phage meta-genome from the USA bioreactor, they found 11 CRISPR spacers with matches to sympatric phage genome fragments. Thus, the CRISPR record of interaction with phage, coupled with geographical sampling, suggests a coevolutionary mosaic that overpowers the effects of dispersal. A similar result was found in a comparative genomic analysis of *Sulfolobus islandicus* CRISPR arrays and spindle-shaped virus (SSV) sequences, where viruses exhibited clear biogeographic structure apparently driven by ongoing adaptation to local host strains (Held & Whitaker 2009).

The importance for coevolution of ecological factors, such as productivity (Lopez-Pascua & Buckling 2008), migration (Morgan *et al*. 2005) and spatial structuring (Morgan *et al*. 2007), has been addressed with experimental evolution in controlled laboratory environments. CRISPR studies such as those described above, which employ structured sampling strategies, potentially allow analogous coevolutionary dynamics to be studied in natural populations. However, a key limitation of this approach is that inferring local adaptation from the presence of spacers and the corresponding phage sequence could be misleading if not all spacers are always expressed (e.g. Brouns *et al*. 2008), or if the CRISPR system is not functional. Metagenomic studies in conjunction with phenotypic assays of phage infectivity on local bacterial hosts would unequivocally determine the level of local adaptation, but in many cases laboratory cultures of microbes are difficult to establish (but see Vos *et al*. 2009).

CRISPR studies have shown that our inability to culture the vast majority of bacteria does not necessarily stop us from accurately inferring coevolutionary dynamics. This is certainly the case for microbial biofilms of thermophilic (*Synechcoccus thermophilus*) or acidophilic (*Leptospirillum*) bacteria and their natural phage pathogens (Tyson & Banfield 2008; Heidelberg *et al*. 2009). Comparative genomics of CRISPR arrays with the viral meta-genomes from these environments demonstrated a history of selective sweeps at a CRISPR locus (Tyson & Banfield 2008) and an abundance of single-nucleotide polymorphisms in the viral sequence corresponding to all but the most recently incorporated spacers (Heidelberg *et al*. 2009). This suggests a scenario of rapid coevolutionary interactions between the microbial hosts and phage that maintains population-wide genetic diversity. It appears that such records of coevolution past may run extremely deep. For example, CRISPR from *Leptospirillum* populations found in acid mine drainage and subaerial biofilms (39°C, pH approx. 1; Andersson & Banfield 2008) were found to contain 37 distinct CRISPR arrays containing a total of 6044 spacer sequences (of which 2348 were unique). Most of these were of viral origin (up to 40 per cent at a single CRISPR locus), although some came from extrachromosomal elements such as plasmids and transposons, indicating that CRISPR loci may, for reasons that are not yet clear, contain records of other types of partnership (Marraffini & Sontheimer 2008).

The studies described above used a spatial sampling strategy to test for local adaptation. Such spatial patterns of local adaptation can indirectly indicate the action of coevolution, but regular and frequent sampling through time offers a more direct window on the process. As with spatial sampling, it would add value to confirm field patterns of phage adaptation with laboratory experimentation, specifically via time-shift experiments, where antagonists are sampled at different time points and controlled infections carried out between them (Gaba & Ebert 2009). By observing the patterns of infectivity among these combinations, one may infer the underlying coevolutionary dynamics (Gandon *et al*. 2008). Time-shift experiments have been achieved, for example, in laboratory studies of *Pseudomonas* bacteria and phage (Buckling & Rainey 2002*b*; Brockhurst *et al*. 2003), and on larger organisms in the field by collecting resting eggs of *Daphnia* and long-living parasite transmission spores from dated lake sediment cores (Decaestecker *et al*. 2007). What has never been achieved is a union of field observation, phenotypic assays and documentation of their molecular underpinnings. One test of parasite local adaptation in a plant–pathogen system (Laine 2007) reported discordant results when measuring parasite fitness in either a field-transplant experiment or a laboratory cross-infection experiment. If CRISPRs are employed to investigate patterns of coevolution across time or space in a bacterial system that is also amenable to laboratory culture, the intersection between laboratory and field-based investigations would provide clear benefits to our understanding of local adaptation and coevolutionary dynamics (Nuismer & Gandon 2008).

Work in some host–pathogen systems has sought to infer the nature of coevolutionary dynamics from the underlying genetics of infection. Most notably, workers have compared the propensity of the ‘gene-for-gene’ and ‘matching allele’ infection models to promote and maintain genetic polymorphism (Agrawal & Lively 2002). However, in conventional models, resistant phenotypes are derived by mutations on the host genome, whereas CRISPR-mediated resistance is explicitly linked to genetic variance in the prevailing pathogen population. It is therefore difficult to predict CRISPR-mediated coevolutionary dynamics without some knowledge of the infection genetics particular to this mechanism. For example, CRISPR-mediated systems suggest exquisite specificity: hosts are no longer protected from phage that acquires a single mutation in spacer-derived sequences. These phages are thus universally infective and should quickly sweep to high frequency. Will this happen so fast that the new phage mutants quickly fix in populations, eroding genetic variation in the process? Or will acquisition of a phage-derived spacer into a CRISPR locus occur quickly enough to halt the march to fixation? It is presently unclear how rapidly populations can acquire resistance via CRISPR; that is, what proportion of infected host cells incorporate viral spacers before lysis occurs.

One may also speculate on the relative importance of CRISPR-based resistance relative to other phage-resistance mechanisms, such as cell-surface receptor modification. Receptor modification offers a first line of defence that impedes the entry of most phages, but mutations at these receptors often come at some metabolic cost to the host, as they are the same receptors involved in nutrient uptake (e.g. Ferenci *et al*. 1980). In addition, viral population sizes and mutation rates are much higher than for their bacterial hosts (Drake *et al*. 1998), so such resistance can in principle be easily evaded. Depending on how efficient spacer acquisition by CRISPRs is, such a second line of defence once phage have entered the host cell could relax the costs associated with receptor modification, and also provide an invaluable chance of halting infection once counter-resistance to receptor modification has occurred. This interplay between CRISPR and other defence mechanisms is yet to be explored, and addressing these issues will tell us much about the genetic constraints on CRISPR-mediated resistance evolution (in particular, the rates at which CRISPRs evolve relative to changes in pathogen-imposed selection over time).

## 4. Measuring the costs of CRISPR-mediated resistance

Coevolutionary dynamics and levels of polymorphism in resistance are tightly linked with the costs associated with resisting infection (Antonovics & Thrall 1994). Costs of resistance are a common feature of most host–parasite systems (Sheldon & Verhulst 1996), and reveal a trade-off (a negative co-variance) between resistance and other life-history traits (e.g. Kraaijeveld & Godfray 1997). If CRISPR-mediated resistance were not costly, we would expect an indefinite accumulation of phage-derived spacers in the host genome. There may be costs associated with CRISPR length, or with the number of CRISPRs on the genome, and such costs may maintain polymorphism in the number and identity of spacers present in the bacterial population. So far, measurements of costs of resistance in wild bacteria–phage systems are limited (Lennon *et al*. 2007), and whether CRISPR is costly owing to acquisition of additional spacers has yet to be tested.

There are several potential ways that CRISPRs might be a costly system. As hosts incorporate more spacers (figure 1), longer CRISPR arrays may simply take longer to replicate, resulting in reduced growth rate. A linked issue is that as CRISPR arrays get longer, the transcription of the repeat sequence separating phage-derived spacers may cause loss of replication fidelity during cell division, similar to what occurs in microsatellites (Bzymek & Lovett 2001). Hence, hosts with long CRISPR arrays might accumulate a larger number of deleterious mutations and, consequently, have lower fitness. Considering how CRISPRs might be costly highlights several unknowns about how this mechanism provides a memory of past infections. How many spacers are expressed during infection? That is, do CRISPR loci allow memory against enemies from the distant past, or are defences maintained only for the most recent pathogenic encounters? Are there physiological costs of expressing many spacers? Does variation in CRISPR array size correlate with the diversity of phage that hosts are exposed to? A recent theoretical exploration of the evolution of gene expression in host–parasite systems (Nuismer & Otto 2005) found that, while coevolution often favours the co-expression of resistance alleles in hosts (maximizing the chances of pathogen recognition), this depends on the underlying infection genetics and on the costs of resistance. While measuring costs of resistance is often challenging (Pease & Bull 1988; Mealor & Boots 2006), if CRISPRs can be incorporated into a laboratory system then we could exploit the practical advantages of experimental evolution with microbes (Jessup *et al*. 2004; Buckling *et al*. 2009) to quantify the mode and strength of selection on CRISPR-mediated resistance in controlled environments.

## 5. Experimental evolutionary epidemiology

Evolutionary biology is, in principle, well poised to understand the processes and mechanisms that underpin changing pathogenicity, with well-established general theories of adaptation (Fisher 1930; Price 1972), invasion dynamics (May *et al*. 2001; Schreiber & Lloyd-Smith 2009) and host–pathogen coevolution (Anderson & May 1982; Best *et al*. 2009; Gandon & Day 2009). But how do evolution and epidemics interact to determine the population burden of disease? A key challenge for future research is to establish an experimental system where CRISPRs may directly inform on viral epidemiological and evolutionary dynamics in natural populations. What is required is a host–pathogen system where it is possible to monitor epidemics regularly and to identify host and pathogen genotypes present during all stages of epidemic onset. Microbial systems where seasonal viral epidemics are known to occur (e.g. Yoshida *et al*. 2008) would appear to be good candidates for such studies, and if CRISPRs are added to the equation, there is the potential for frequent field sampling to reveal the genetic details of epidemic onset. The advantage of CRISPRs would be in providing a high-resolution genetic marker that recapitulates the history of infections in the host genome and allows individual phage strains to be tracked by following their presence on these same host genomes. With this information in hand, it is then possible to begin addressing specific questions about the dynamics of adaptation in the context of epidemic spread: does pathogen adaptation occur mainly from standing genetic variation or from novel mutation (Hermisson & Pennings 2005; Barrett & Schluter 2008)? What effect do bottlenecks have on the contributions of selection or drift during pathogen emergence (Dlugosch & Parker 2008)? Is there clonal interference between invading strains during epidemic onset (Miralles *et al*. 1999; Pepin & Wichman 2008), and how does recombination between them affect the epidemiological dynamics (Colegrave 2002; Cooper 2007)?

While the existence of CRISPR arrays in prokaryotes was originally recognized in the late 1980s (Ishino *et al*. 1987), appreciation of their role in mediating resistance against viral pathogens is very recent. The metagenomic-based CRISPR studies described here have already demonstrated the potential to observe bacteria and phage coevolution in their natural settings and in real time, but many opportunities for fieldwork remain. Characterizing this mechanism in host–pathogen systems that are known to coevolve and are amenable to laboratory life (e.g. Vos *et al*. 2009) will further expand the range of questions that can be explored, but the key opportunity would seem to be the possibility of detailed analysis of coevolution in the wild. The molecular book-keeping of the CRISPR system may thus help unravel the nature of the coevolutionary process under natural conditions of temporal and spatial environmental heterogeneity.
